# S100A12 Expression Is Modulated During Monocyte Differentiation and Reflects Periodontitis Severity

**DOI:** 10.3389/fimmu.2020.00086

**Published:** 2020-01-31

**Authors:** Ronaldo Lira-Junior, Sofia Björnfot Holmström, Reuben Clark, Stephanie Zwicker, Mirjam Majster, Gunnar Johannsen, Björn Axtelius, Sigvard Åkerman, Mattias Svensson, Björn Klinge, Elisabeth A. Boström

**Affiliations:** ^1^Division of Oral Diseases, Department of Dental Medicine, Karolinska Institutet, Huddinge, Sweden; ^2^Department of Oral Diagnostics, Faculty of Odontology, Malmö University, Malmö, Sweden; ^3^Department of Orofacial Pain and Jaw Function, Faculty of Odontology, Malmö University, Malmö, Sweden; ^4^Department of Medicine, Center for Infectious Medicine, Karolinska Institutet, Huddinge, Sweden; ^5^Department of Periodontology, Faculty of Odontology, Malmö University, Malmö, Sweden

**Keywords:** monocytes, monocyte-derived cells, S100A12 protein, periodontal diseases, gingiva, saliva

## Abstract

S100A12 is a calcium-binding protein of the S100 subfamily of myeloid-related proteins that acts as an alarmin to induce a pro-inflammatory innate immune response. It has been linked to several chronic inflammatory diseases, however its role in the common oral immunopathology periodontitis is largely unknown. Previous *in vitro* monoculture experiments indicate that S100A12 production decreases during monocyte differentiation stages, while the regulation within tissue is poorly defined. This study evaluated S100A12 expression in monocyte subsets, during monocyte-to-macrophage differentiation and following polarization, both in monoculture and in a tissue context, utilizing a three-dimensional co-culture oral tissue model. Further, we explored the involvement of S100A12 in periodontitis by analyzing its expression in peripheral circulation and gingival tissue, as well as in saliva. We found that S100A12 expression was higher in classical than in non-classical monocytes. S100A12 expression and protein secretion declined significantly during monocyte-to-macrophage differentiation, while polarization of monocyte-derived macrophages had no effect on either. Peripheral monocytes from periodontitis patients had higher S100A12 expression than monocytes from controls, a difference particularly observed in the intermediate and non-classical monocyte subsets. Further, monocytes from periodontitis patients displayed an increased secretion of S100A12 compared with monocytes from controls. In oral tissue cultures, monocyte differentiation resulted in increased S100A12 secretion over time, which further increased after inflammatory stimuli. Likewise, S100A12 expression was higher in gingival tissue from periodontitis patients where monocyte-derived cells exhibited higher expression of S100A12 in comparison to non-periodontitis tissue. In line with our findings, patients with severe periodontitis had significantly higher levels of S100A12 in saliva compared to non-periodontitis patients, and the levels correlated to clinical periodontal parameters. Taken together, S100A12 is predominantly secreted by monocytes rather than by monocyte-derived cells. Moreover, S100A12 is increased in inflamed tissue cultures, potentially as a result of enhanced production by monocyte-derived cells. This study implicates the involvement of S100A12 in periodontitis pathogenesis, as evidenced by increased S100A12 expression in inflamed gingival tissue, which may be due to altered circulatory monocytes in periodontitis.

## Introduction

Monocytes are mononuclear phagocytes, which after birth derive from hematological precursors in the bone marrow and enter the blood circulation (1). In blood, human monocytes are divided into three main subsets based on the expression of CD14 and CD16. Classical monocytes have high CD14 expression and no CD16 (CD14^hi^CD16^−^), intermediate monocytes show high CD14 and low CD16 (CD14^hi^CD16^+^), and non-classical monocytes have low expression of CD14 together with high CD16 (CD14^+^CD16^hi^) (2). These subsets present different transcriptional profiles with CD16^+^ monocytes being at a more advanced stage of myeloid differentiation (3, 4). The intermediate subset has been suggested to correspond to an activation and/or differentiation state of CD14^+^ monocytes (5), though a closer relationship to the non-classical subset has been reported (6).

Following injury, infection or inflammation, monocytes are rapidly recruited to the tissues where they become activated, alter their phenotype and can mature into inflammatory macrophages. This monocyte-to-macrophage transition occurs in a non-synchronized manner, and these cells are highly heterogeneous in the tissue (1). *In vitro*, macrophage diversity has been functionally classified into two groups: “classically activated” (M1) or “alternatively activated” (M2) macrophages. Macrophages undergo activation to M1 or M2 phenotype in response to toll-like receptor (TLR) ligands/interferon (IFN)-γ or interleukin (IL)-4/IL-13, respectively. M1 macrophages are generally efficient producers of reactive oxygen and nitrogen intermediates and inflammatory cytokines, while M2 macrophages have high levels of scavenger, mannose and galactose-type receptors, being fundamental for tissue remodeling, repair and healing (7). In tissue however, a plethora of stimuli occurs at the same time, inducing a spectrum of activation rather than the black and white scenario that occurs with M1/M2 polarization *in vitro*.

S100A12 is a member of the S100 family of low molecular weight proteins, which are characterized by two calcium binding EF-hand motifs connected by a central hinge region (8, 9). S100A12 is mainly expressed in the cytoplasm of myeloid cells and, upon release, acts as a proinflammatory alarmin (9, 10). Interestingly, its gene expression is higher in classical than in non-classical monocytes (3, 6), and it decreases during monocyte to macrophage maturation (11). S100A12 binds to receptor for advanced glycation end products (RAGE) and TLR4, it is chemotactic for leukocytes and induces a strong inflammatory response in monocytes (12, 13). Its expression is markedly increased at sites of inflammation, and the levels in circulation might be useful as a measure of disease activity in chronic inflammatory diseases (14–17).

Periodontitis is a chronic inflammatory disease characterized by destruction of the tooth-supporting structures, which can lead to tooth loss and contribute to the systemic inflammatory burden (18). Its pathogenesis involves a complex interplay between the dysbiotic microbiota and the host immune-inflammatory response (19), where myeloid cells play a critical role in the periodontal injury. These cells infiltrate the gingival tissue during disease, undermine oral mucosa integrity through the production of matrix metalloproteinase (MMP)-12 (20) and contribute to the alveolar bone resorption (21). It has been shown that S100A12 levels in saliva are associated with periodontal disease (22). However, the role of monocyte-produced S100A12 in periodontal inflammation has not been investigated yet.

Therefore, the present study aimed to evaluate S100A12 dynamics during monocyte-to-macrophage differentiation and polarization. Further, we aimed to explore its involvement in periodontitis using a 3D co-culture tissue model, and by analyzing S100A12 expression in tissue, peripheral circulation, and saliva from patients with periodontitis.

## Materials and Methods

### Human Subjects

Healthy participants and patients with periodontitis were recruited at the Department of Dental Medicine, Karolinska Institutet, Stockholm, and at the specialist clinic Danakliniken, Danderyd, Sweden. Periodontitis patients were included based on the presence of at least 4 teeth with probing pocket depths ≥ 6 mm and radiographic bone loss. Control participants had no periodontal pockets ≥ 4 mm and no history of periodontitis. Participants were excluded if they had chronic inflammatory diseases that could affect the periodontium, or had used antibiotics or corticosteroids 3 months before the inclusion. Additionally, a cohort of individuals living in the Kalmar county, Sweden, was recruited. They were randomly selected, orally examined and provided a stimulated saliva sample. Participants answered a questionnaire containing data about age, sex, smoking habits, and presence of diseases. Smoking was recorded as current smokers or never/former smokers.

This study was carried out in accordance with the recommendations of the regional ethics committee in Stockholm, Sweden and of the regional ethics committee at the Lund University, Sweden with written informed consent from all subjects. All subjects gave written informed consent in accordance with the Declaration of Helsinki. The protocol was approved by the regional ethics committee in Stockholm, Sweden (Dnr. 2012/1579-32 and 2017/1333-32) and by the regional ethics committee at the Lund University, Sweden (Dnr. 2011/366).

### Monocyte Isolation, Differentiation, and Polarization

Buffy-coated blood from anonymous healthy donors were used for the *in vitro* experiments. To study monocytes in periodontitis, peripheral blood was also collected in EDTA-containing vacutainers from periodontitis patients and periodontally healthy individuals. Peripheral blood mononuclear cells (PBMCs) were isolated using Ficoll-Hypaque gradient centrifugation (BD Diagnostics, San Jose, CA, USA), after which monocytes were isolated using the EasySep Human monocyte enrichment kit without CD16 depletion (StemCell Technologies, Vancouver, BC, Canada), according to the manufacturer's instructions. Monocytes from healthy donors were cultured in 6-well plates (5 × 10^5^ monocytes/well) in complete RPMI 1640 medium (10 mM HEPES, 2 mM L-glutamine, 100 U/ml penicillin, 100 mg/ml streptomycin, and 10% FCS) supplemented with CSF-1 (50 ng/ml; BioLegend, San Diego, CA, USA) for 1, 3, and 8 days at 37°C, 5% CO_2_, to assess the monocyte-to-macrophage differentiation. After 8 days in culture, macrophages were polarized with LPS (50 ng/ml; Sigma-Aldrich, St. Louis, MO, USA) and IFN-γ (50 ng/ml; BioLegend, San Diego, CA, USA) or IL-4 and IL-13 (50 ng/ml each; BioLegend, San Diego, CA, USA) for another 24 h. Non-polarized macrophages were used as controls. PBMCs from periodontitis patients and periodontally healthy individuals were stored frozen after collection. The PBMCs where thawed in complete RPMI, and used for flow cytometry staining or monocyte isolation followed by *in vitro* culture. The monocytes were cultured (37°C, 5% CO_2_) in 24-well plates in complete RPMI with CSF-1 (50 ng/ml; Biolegend, San Diego, CA, USA) at a concentration of 3 × 10^5^ cells/ml and incubated for 24 h.

### 3D Oral Tissue Culture

A 3D oral tissue model was set up containing epithelial cells (TERT-immortalized normal human oral keratinocyte line OKF6/TERT-2, kindly provided by J. Rheinwald) (23), primary fibroblasts (24), and monocytes as previously described (20). Briefly, 3-μm pore size transwell inserts were placed in 6-well plates and coated with a mixture of bovine type I collagen (PureCol, Cell Systems, Troisdorf, Germany) and DMEM (GE Healthcare Life Sciences, Uppsala, Sweden). Fibroblasts (7.5 × 10^4^ cells/model) were suspended in complete DMEM and diluted in a PureCol and DMEM suspension with addition of media after 2 h and cultured for 7 days in 5% CO_2_ at 37°C. Monocytes (4 × 10^5^/model) in complete RPMI were then added on top of the fibroblast layer and incubated for 1.5 h in 5% CO_2_ at 37°C, after which complete DMEM was added for a 24 h incubation. Epithelial cells (4 × 10^5^/model) in complete K-SFM were added on top of the fibroblast and monocyte layers. After a 1.5 h incubation in 5% CO_2_ at 37°C, complete K-SFM was added for a 48 h incubation. The models were air-exposed by removing the media, followed by the addition of complete K-SFM supplemented with an additional 0.3 mM CaCl_2_ only to the outer chamber. To assess time-dependent secretion of S100A12, models were cultured for 3, 5, and 7 days after monocytes were implanted and supernatants were collected. Similar models without the addition of monocytes were also set up.

Oral tissue cultures were repeatedly stimulated with K-SFM containing *E. coli* LPS (100 ng/ml; Invivogen, San Diego, CA, USA) and IFN-γ (50 ng/ml; BioLegend, San Diego, CA, USA). Complete K-SFM only was used as a control. The stimulations were added in the upper compartment at day 2, 3, and 5 after monocyte implantation in conjunction with media change in the bottom compartment. Supernatants were collected at day 7 after monocyte implantation. Additionally, conditioned medium from oral tissue models without monocytes were used to stimulate monocytes and supernatants were collected after 24 h.

### Real-Time PCR

RNA from *in vitro* cultured cells was isolated using the Quick-RNA MiniPrep kit (Zymo Research Corp, Irvine, CA, USA), and reverse transcribed using the High Capacity cDNA Reverse Transcription Kit (Applied Biosystems, Foster City, CA, USA) according to the manufacturer's instructions. S100A12 mRNA expression was determined using SYBR Green (Bio-Rad Laboratories, Hercules, CA, USA) in the 7500-fast-real-time detection system (Applied Biosystems, Foster City, CA, USA) using specific primers (forward: CACATTCCTGTGCATTGAGG/reverse: GGTGTCAAAATGCCCCTTC, Eurofins, Ebersberg, Germany) related to the housekeeping gene GAPDH (forward: TCCACTGGCGTCTTCACC/reverse: GGCAGAGATGATGACCCTTTT) by the ΔΔCt method.

### Gingival Tissue Collection and Digestion

Gingival tissue was harvested during periodontal surgery from an inflamed site with a persistent periodontal pocket ≥6 mm in periodontitis patients (*n* = 6). In the controls, gingival tissue was harvested from a site without periodontal pocket in conjunction with an implant surgery or tooth extraction performed for reasons other than periodontitis (*n* = 6). Tissues were placed in complete RPMI 1640 media (GE Healthcare Life Sciences, Uppsala, Sweden). The tissues were cut into small pieces and incubated with 0.3 mM CaCl_2_ in phosphate buffered saline (PBS) at 37°C with magnetic stirring for 10 min. After that, they were incubated with Collagenase II (0.5 mg/ml; Sigma-Aldrich, St. Louis, MO, USA) and DNase I (0.25 mg/ml; Roche, Basel, Switzerland) in RPMI 1640 without FCS at 37°C with magnetic stirring for 40 min. The suspension was filtered through a 70-μm mesh filter (Falcon, Corning, NY, USA), washed with complete RPMI, and centrifuged at 400 g for 5 min. Cell suspensions were then stained and analyzed by flow cytometry.

### Immunohistochemistry and Immunofluorescence

Gingival tissue from periodontitis patients (*n* = 4) and healthy controls (*n* = 5) were harvested during periodontal surgery and immediately placed in tissue transport solution (Histocon; Histolab Products AB, Gothenburg, Sweden), after which they were embedded in optimal cutting temperature compound (OCT; Histolab Products AB, Gothenburg, Sweden). 3D oral tissue cultures were incubated in 2M sucrose for 1 h, and then the membranes and attached models were removed from the insert and embedded in OCT. The embedded tissues and 3D cultures were stored at −80°C until sectioning.

Seven micrometers of sections were fixed in acetone and endogenous peroxidase was blocked in methanol and H_2_O_2_. Avidin and biotin solutions (Vector Laboratories, Burlingame, CA, USA) and the appropriate serum were used for blocking of the sections. Tissues were incubated with a monoclonal anti-S100A12 antibody (clone OTI1D1, ThermoFisher, Waltham, MA, USA), a monoclonal anti-CD68 antibody (clone PG-M1, Dako, Glostrup, Denmark) or their respective isotype controls (Abcam, Cambridge, UK) overnight at 4°C. An incubation with the biotinylated secondary antibody (Vector Laboratories, Burlingame, CA, USA) was performed, followed by the ABC complex (Vector Laboratories, Burlingame, CA, USA), and then developed in diaminobenzidine (DAB) solution (Vector Laboratories, Burlingame, CA, USA). Slides were dehydrated, mounted with Pertex (Histolab Products AB, Gothenburg, Sweden) and visualized and photographed using an Olympus BX43 light microscope equipped with an Olympus SC50 camera (Olympus Corporation, Tokyo, Japan).

Double labeling was performed on gingival tissue using the following antibodies: mouse anti-S100A12 (clone OTI1D1) and rat anti-CD206 (clone 309210, R&D Systems, Minneapolis, MN, USA) or isotype controls. Specific staining was detected by flourochrome-labeled goat anti-mouse (AF647) and anti-rat (AF488) antibodies. Cell nuclei counterstain was performed with DAPI (4′,6-Diamidino-2-Phenylindole, Dihydrochloride, ThermoFisher, Waltham, MA, USA) and sections were mounted in Prolong Gold Antifade Mountant (ThermoFisher, Waltham, MA, USA). Immunofluorescence staining was visualized using a Nikon Eclipse E600 Fluorescence Microscope equipped with the Nikon digital camera DMX1200 (Nikon, Tokyo, Japan).

### Western Blot

Total protein from gingival tissue from periodontitis patients (*n* = 6) and controls (*n* = 6) was isolated using a protein extraction buffer (T-Per, ThermoFisher, Waltham, MA, USA) supplemented with a protease inhibitor cocktail (Roche Molecular Systems Inc., Pleasanton, CA, USA). Fifteen μg of total protein per lane was separated by gradient Mini-PROTEAN® TGX™ 4–20% gels (Bio-Rad Laboratories, Hercules, CA, USA) and then transferred to nitrocellulose membranes (GE Healthcare Life Science, Uppsala, Sweden). After incubation with blocking buffer [Tris buffered saline, 0.1% Tween 20 (TBS-T) and 5% non-fat milk powder] for 1 h at RT, membranes were washed with TBS-T and incubated with the S100A12 antibody (clone OTI1D1, ThermoFisher, Waltham, MA, USA) overnight at 4°C. Membranes were then incubated with the secondary horseradish-peroxidase-conjugated IgG (Cell Signaling Technology, Inc., Beverly, MA, USA) at RT for 1 h. As a loading control, β-actin (Cell Signaling Technology, MA, USA) was used. Membranes were developed with Amersham ECL select Western Blotting Detection Reagent (GE Healthcare Life Science, Uppsala, Sweden). The blots were visualized and quantified with the ChemiDoc™ XRS (Bio-Rad Laboratories Inc., Hercules, CA, USA).

### Flow Cytometry

Cell preparations from blood or digested tissues, were incubated with BD Horizon Fixable Viability Stain 510 (BD Bioscience, Franklin Lakes, NJ, USA) diluted in PBS. The cells were then incubated with human Trustain FcX (BioLegend, San Diego, CA, USA) in FACS buffer (2% FCS, 1 mM EDTA in PBS) for 10 min in RT followed by the addition of fluorochrome-conjugated antibodies and an additional 30 min incubation on ice. The following antibodies were used in different combinations: CD14 (clone M5E2, PerCP; BioLegend), CD14 (clone MϕP9, APC; BD Bioscience), CD16 (clone 3G8, BV421; BioLegend), CD90 (clone 5E10, PE; BioLegend), CD45 (clone HI30, BV421; BioLegend), HLA-DR (clone L243, APC or PE/Cy7; BioLegend), CD64 (clone 10.1, PE/Cy7; BioLegend), and CD206 (clone 15-2, APC/Cy7; BioLegend). Cells were fixed and permeabilized with BD Cytofix/Cytoperm, after which they were incubated with the following intracellular antibodies for 30 min on ice: S100A12 (clone 161205, AF488) or IgG2b (AF488), both from R&D Systems (Minneapolis, MN, USA). Cells were analyzed with BD FACSVerse™ (BD Bioscience). Data analysis was performed using the FlowJo software, version 10 (Tree Star, Ashland, OR, USA).

### Plasma Collection

The blood collected from periodontitis patients (*n* = 5) and controls (*n* = 5) was diluted with equal amount of PBS followed by a Ficoll-Hypaque gradient centrifugation (BD Diagnostics). The upper layer was collected and stored at −80°C until analysis.

### Saliva Collection

A clinical oral examination was performed in 336 individuals (51.4 [±17.7] years; 50.6% women) including plaque index, bleeding on probing (BOP) and probing pocket depth (PPD). The degree of alveolar bone loss was determined through radiographic examination. The association between the different periodontal parameters and S100A12 levels in saliva was evaluated by categorizing the patients within the following groups: BOP ≤ 20% or >20% (22); presence or not of pathological periodontal pockets (PPD ≥ 4 mm); and loss of supporting bone tissue <1/3 of the root length (PD-group), ≥1/3 of the root length in <30% of the sites (PD group), and horizontal bone loss ≥1/3 of the root length in >30% of the sites (PD+ group) (25). In addition, the participants were diagnosed according to the new classification of periodontal diseases (26, 27). Stimulated saliva samples were collected during 5 min of chewing on 0.5 g paraffin and the salivary flow was measured. Saliva was immediately frozen at −20°C and then centrifuged and supernatants were stored at −80°C until analysis. Total amount of protein was determined by the Bradford assay.

### ELISA

S100A12 levels in supernatants, plasma, and saliva were analyzed by enzyme-linked immunosorbent assay (R&D Systems, Minneapolis, MN, USA) according to the manufacturer's instructions. The assay range of detection is 7.81–500 pg/ml. The assay was previously validated for saliva by performing spike and recovery tests (22). Saliva and plasma samples were diluted 1:1,000 and 1:5, respectively. Readings were made using a microplate spectrophotometer at 450 nm with wavelength correction set to 540 nm to subtract background (SpectraMAX 340, Sunnyvale, CA, USA).

### Statistical Analysis

Data analysis was performed with GraphPad Prism, version 8 (GraphPad Software, La Jolla, CA, USA). Group comparisons were performed using Mann–Whitney, Kruskal–Wallis or Friedman test with a Dunn's post-test when appropriate. Correlations were analyzed by Spearman rank correlation coefficient. Statistical significance was set at *p* ≤ 0.05.

## Results

### S100A12 Expression Is Higher in Classical Monocytes and Decreases During Monocyte-to-Macrophage Differentiation

**Figure 1:**
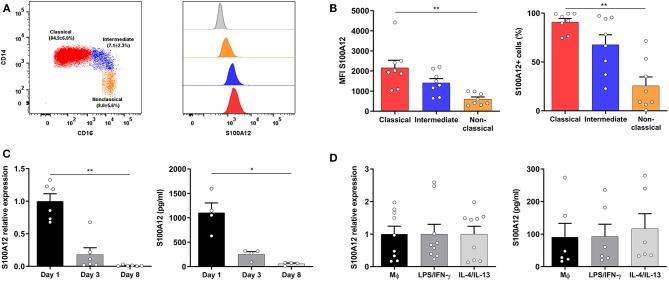
S100A12 expression and secretion during monocyte-to-macrophage differentiation and polarization. **(A)** Representative dot plot showing the gating strategy of human monocyte subsets based on CD14 and CD16 expression after doublet and dead cells exclusion, and HLA-DR expression, as well as mean (±SD) frequency of monocyte subsets. Histogram depicting the S100A12 median fluorescence intensity (MFI) in classical (red), intermediate (blue), and non-classical monocytes (orange), as well as in the isotype control (gray histogram). **(B)** S100A12 MFI and percentage of S100A12^+^ cells in classical, intermediate, and non-classical monocytes (*n* = 8). **(C)** S100A12 relative mRNA expression (normalized to GAPDH) and secretion in monocytes cultured with CSF-1 for 1, 3, and 8 days (*n* = 4–6). **(D)** S100A12 relative mRNA expression (normalized to GAPDH) and secretion in macrophages polarized by LPS/IFN-γ and IL-4/IL-13 for 24 h (*n* = 6–9). Data are presented as mean ± SEM. Differences were calculated with Friedman test with Dunn's *post-hoc* test, *p < 0.05, **p < 0.001.

To gain insight into S100A12 modulation in monocytes and macrophages, we assessed its expression in human monocyte subsets (Figure 1A) and the results showed that classical monocytes displayed higher expression and were more frequently positive for S100A12 than non-classical monocytes (Figure 1B). Next, S100A12 expression and secretion during monocyte-to-macrophage differentiation in culture with CSF-1 for 8 days were evaluated and we found that both mRNA expression and protein secretion were significantly higher in monocytes (day 1) than in macrophages (day 8). No significant difference in mRNA expression or protein secretion was observed in comparison with day 3. In fact, S100A12 secretion was on average 19 times higher in monocytes in comparison with macrophages (Figure 1C). Moreover, when macrophages were classically (LPS/IFN-γ) or alternatively activated (IL-4/IL13) no significant difference was seen in the expression and secretion of S100A12 between unpolarized and “M1” or “M2” subsets (Figure 1D).

### S100A12 Expression Is Altered in Circulating Monocytes From Periodontitis Patients

**Figure 2:**
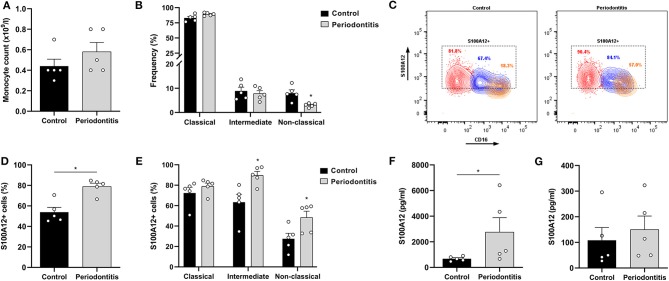
S100A12 expression in peripheral monocytes from periodontitis patients and healthy controls. **(A)** Monocyte counts (x10^9^/l) in participants with periodontitis (*n* = 5) and controls (*n* = 5). **(B)** Frequency of classical, intermediate, and non-classical monocytes in participants with periodontitis (*n* = 5) and controls (*n* = 5). **(C)** Representative contour plots of S100A12^+^ classical (red), intermediate (blue), and non-classical (orange) monocytes in periodontitis and control. Proportion of S100A12^+^ cells in each subset is included in the plot. **(D)** Percentage of S100A12^+^ monocytes in periodontitis patients (*n* = 5) and controls (*n* = 5). Monocytes were identified based on the expression of CD14, CD16, and HLA-DR. **(E)** Percentage of S100A12^+^ cells in classical, intermediate, and non-classical monocytes from periodontitis patients (*n* = 5) and controls (*n* = 5). **(F)** Secretion of S100A12 in monocytes from periodontitis patients (*n* = 5) and controls (*n* = 5) after 24 h in culture. **(G)** S100A12 levels in plasma from periodontitis patients (*n* = 5) and controls (*n* = 5). Data are presented as mean ± SEM. Differences were calculated with Mann–Whitney test, *p < 0.05.

Previous studies have reported an increase in the proportion of non-classical monocytes in periodontitis (28, 29). We investigated whether peripheral monocytes from periodontitis patients (52.6 ± 5.1 years old; 2 females and 3 males) present differential S100A12 expression and secretion when compared with monocytes from periodontally healthy participants (42.4 ± 15.9 years old; 3 females and 2 males), and whether this difference was related to a particular subset. The monocyte counts did not differ significantly between periodontitis patients and controls (Figure 2A). However, the frequency of non-classical monocytes was lower in periodontitis patients (Figure 2B). The classical subset displayed a tendency toward higher frequency in periodontitis (*p* = 0.055). Mean counts (±SD) of the classical, intermediate, and non-classical subsets in the controls were 3.9 (±1.4), 0.4 (±0.1), and 0.3 (±0.1) × 10^8^/L, respectively. In periodontitis, they were 5.2 (±1.9), 0.4 (±0.1), and 0.2 (±0.1) × 10^8^/L. The percentage of S100A12^+^ monocytes was higher in periodontitis than in controls (Figures 2C,D). In comparison to healthy controls, a greater proportion of intermediate and non-classical monocytes from periodontitis patients were S100A12^+^ (Figure 2E). Classical monocytes in periodontitis patients were more frequently S100A12^+^, although this did not reach significance (*p* = 0.055). After 24 h in culture, monocytes from periodontitis patients secreted significantly higher levels of S100A12 than control monocytes (Figure 2F). However, plasma levels of S100A12 did not differ significantly between the groups (Figure 2G).

### S100A12 Secretion Is Increased by Inflammatory Stimuli in 3D Oral Tissue Cultures

**Figure 3:**
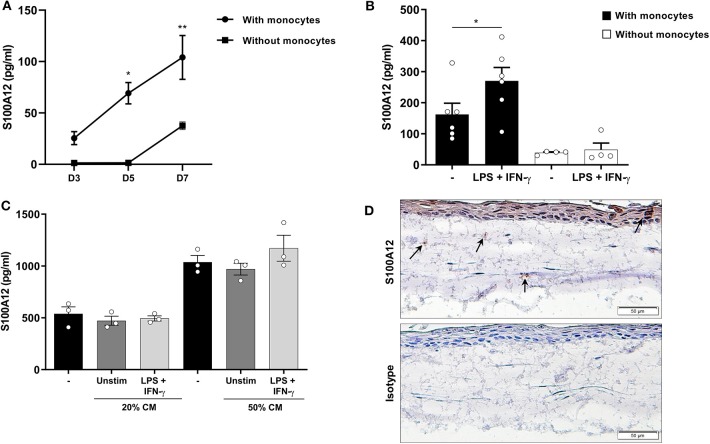
Secretion of S100A12 in a 3D oral tissue culture with and without monocytes after inflammatory stimulation. **(A)** Time-dependent secretion of S100A12 in cultures with (*n* = 4) and without healthy human monocytes (*n* = 3) after 3, 5, and 7 days in culture. **(B)** S100A12 secretion on day 7 after monocyte implantation after repeated stimulation with LPS and IFN-γ in cultures with (*n* = 6) and without monocytes (*n* = 4). **(C)** S100A12 secretion by monocytes (*n* = 3) after 24 h stimulation with conditioned medium (CM) from cultures without monocytes (*n* = 3). **(D)** Immunohistochemical staining of S100A12 or isotype control in an oral tissue culture with monocytes. Arrows indicate S100A12^+^ cells. Data are presented as mean ± SEM. Differences were calculated with Wilcoxon test or Friedman test with Dunn's *post-hoc* test, *p < 0.05, **p < 0.001.

To investigate the modulation of S100A12 production by monocytes in a setting associated with tissue inflammation, we used a 3D oral tissue culture resembling the oral tissue containing epithelial cells, fibroblasts, and monocytes from healthy volunteers. CD68^+^ cells were present in the cultures showing the engraftment and differentiation of monocytes (Supplementary Figure 1). Oral tissue cultures with monocytes showed significantly higher S100A12 levels than those without (*p* < 0.0001). Further, a significant time-dependent increase in the secretion of S100A12 was seen in the cultures with monocytes, however no significant difference was found in cultures without monocytes (Figure 3A). When stimulated with a combination of LPS and IFN-γ there was a significant increase in S100A12 levels in cultures with monocytes, whereas no significant difference was seen in cultures without (Figure 3B). To assess whether factors produced by epithelial cells and/or fibroblasts would lead to S100A12 secretion by monocytes, we stimulated them with conditioned medium from cultures without monocytes, however no significant effect was observed (Figure 3C), which suggests S100A12 secretion might be dependent on direct interaction within the model. A representative immunohistochemical analysis showed S100A12 staining in a 3D oral tissue culture with monocytes (Figure 3D).

### S100A12 Expression Is Higher in Gingival Tissue Affected by Periodontitis

**Figure 4:**
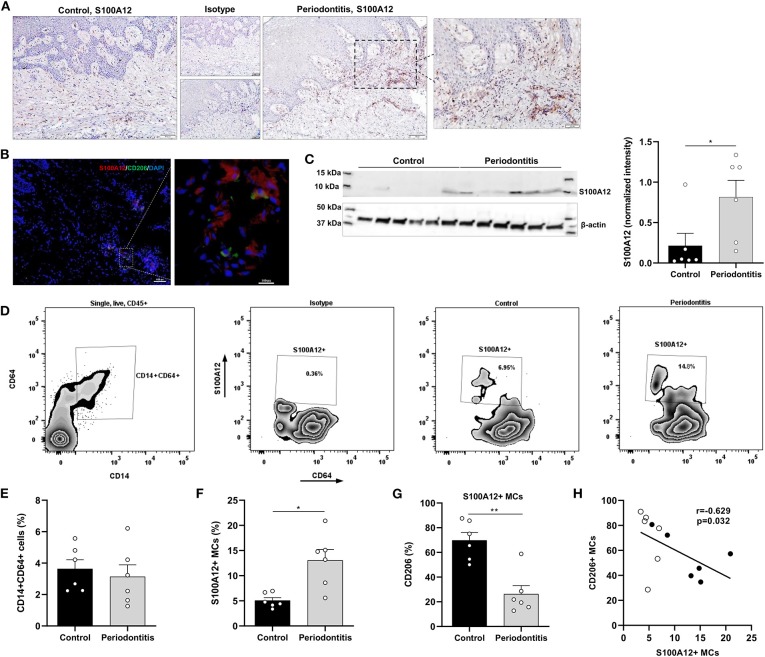
S100A12^+^ monocyte-derived cells in gingival tissue from periodontitis patients and healthy controls. **(A)** Representative immunohistochemical staining of S100A12 in gingival tissue from a periodontitis patient and control. **(B)** Immunofluorescent staining of gingival tissue from periodontitis patient showing the presence of S100A12 (red) and CD206 (green). Nuclei was counterstained with DAPI (blue). **(C)** Western blot analysis of S100A12 monomer in gingival tissue from periodontitis patients (*n* = 6) and controls (*n* = 6), and S100A12 expression presented as normalized intensity to β-actin. **(D)** Representative zebra plot showing the gating strategy of monocyte-derived cells (MCs) in gingival tissue based on CD14 and CD64 expression on single, live, CD45^+^ cells, and zebra plots depicting the S100A12^+^ MCs in health and in periodontitis. Proportion of S100A12^+^ cells is included in the plot. **(E)** Percentage of CD14^+^CD64^+^ cells within the CD45^+^ compartment in gingival tissue from periodontitis patients (*n* = 6) and controls (*n* = 6). **(F)** Percentage of S100A12^+^ cells within the CD14^+^CD64^+^ compartment in gingival tissue from periodontitis patients (*n* = 6) and controls (*n* = 6). **(G)** Percentage of CD206^+^ cells in S100A12^+^ MCs in gingival tissue from periodontitis patients (*n* = 6) and controls (*n* = 6). **(H)** Spearmen correlation between the percentage of S100A12^+^ MCs and the percentage of CD206^+^ MCs. White and black circles indicate controls and periodontitis participants, respectively. Data are presented as mean ± SEM. Differences were calculated with Mann–Whitney test, *p < 0.05, **p < 0.001.

Since inflammatory stimuli modulates S100A12 levels in oral tissue cultures, the expression of S100A12 in gingival tissue affected by periodontitis was evaluated. Immunohistochemical analysis showed strong S100A12 staining in the connective tissue mainly in conjunction with the inflammatory infiltrate in periodontitis, whereas in the controls a weak staining was seen in the connective tissue (Figure 4A). The presence of CD68^+^ cells in gingival tissue is presented in a Supplementary Figure 1. Immunofluorescence staining of gingival tissue showed S100A12 expression can be seen in proximity of CD206-expressing cells (Figure 4B). Western blot evaluation of tissue protein extracts revealed a significantly higher expression of S100A12 in periodontitis compared to controls (Figure 4C).

Further, S100A12^+^ monocyte-derived cells (MCs) in gingival tissue from periodontitis patients and controls were characterized. Digested tissues were stained and live, single, CD45^+^ cells expressing CD14, and CD64 were gated (Figure 4D). These cells also expressed HLA-DR. No significant difference in the proportion of CD14^+^CD64^+^ cells was observed between periodontitis and control participants (Figure 4E). However, a significant increase in the proportion of S100A12^+^ MCs was found in periodontitis (Figure 4F), and the periodontitis-associated S100A12^+^ MCs were less frequently CD206^+^, a marker of alternatively activated macrophages (Figure 4G). A significant, negative correlation was seen between the proportion of S100A12^+^ and CD206^+^ cells in gingival tissue (Figure 4H).

### Salivary Levels of S100A12 Are Elevated in Severe Periodontitis

**Figure 5:**
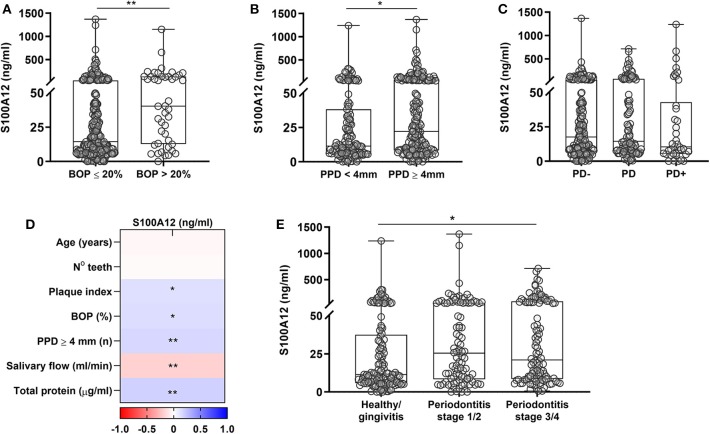
Salivary levels of S100A12 in relation to periodontal disease. **(A)** S100A12 levels in saliva from participants having bleeding on probing (BOP) ≤ 20% (*n* = 292) and those having BOP > 20% (*n* = 44). **(B)** Salivary levels of S100A12 in participants having (*n* = 201) or not probing pocket depth (PPD) ≥ 4 mm (*n* = 135). **(C)** Levels of S100A12 in saliva from participants without bone loss (PD–; *n* = 175), with localized bone loss (PD; *n* = 103), and generalized bone loss (PD+; *n* = 44). Fourteen radiographs were missing. **(D)** Spearman correlation heat map between S100A12 and clinical parameters. **(E)** Salivary levels of S100A12 in healthy/gingivitis participants (*n* = 133) and those with periodontitis stages I/II (*n* = 95) or stages III/IV (*n* = 108). Data are presented as median and quartiles. Differences were calculated with Mann–Whitney test or Kruskal–Wallis with Dunn's *post-hoc* test, *p < 0.05, **p < 0.001.

Lastly, the levels of S100A12 in saliva from a large cohort of orally examined participants (*n* = 336) were measured to investigate whether S100A12 is a potential biomarker for periodontal disease. Each participant was diagnosed according to the new classification of periodontal diseases (26, 27). Clinical characteristics are presented in Table 1. Participants having periodontitis stages III/IV were significantly older than healthy/gingivitis and periodontitis stages I/II participants. No significant difference was observed among the groups regarding the frequency of smokers. Participants with periodontitis stages III/IV had the worst periodontal status as evidenced by lower number of teeth and increased plaque index, bleeding, and number of periodontal pockets. Comparing the levels of S100A12, participants with BOP > 20% showed significantly higher levels than those with BOP ≤ 20% (Figure 5A). The presence of PPD ≥ 4 mm was also related to significantly increased levels of S100A12 in saliva (Figure 5B), whereas no significant difference was seen based on the amount of bone loss (Figure 5C). S100A12 correlated positively with plaque index, BOP, the number of PPD ≥ 4 mm, and the total amount of protein. On the contrary, it correlated negatively with the salivary flow rate (Figure 5D). S100A12 levels were significantly increased in patients with more severe stages of periodontitis (III and IV) in comparison with healthy/gingivitis participants. Patients having periodontitis stages I and II exhibited a tendency toward higher S100A12 levels than the healthy/gingivitis individuals (*p* = 0.06; Figure 5E).

**Table 1:** Clinical characteristics of the study groups.

	**Healthy/gingivitis (*n* = 133)**	**Periodontitis** **stage I/II** **(*n* = 95)**	**Periodontitis** **stage III/IV** **(*n* = 108)**	*p*-value^*^
Age (years)	45.9 (±19.1)	47.7 (±15.1)	61.5 (±13.3)^a^^,^^b^	<0.001
Males, *n* (%)	55 (41.4)	50 (52.6)	61 (56.5)	0.041
Females, *n* (%)	78 (58.6)	45 (47.4)	47 (43.5)	
Smokers, *n* (%)	15 (11.3)	12 (12.6)	17 (15.7)	0.596
N° teeth	25.5 (±4.2)	26.3 (±2.6)	24.0 (±4.0)^a^^,^^b^	<0.001
Plaque index (%)	18.9 (±26.0)	17.1 (±16.8)	22.4 (±23.1)^a^	0.011
BOP (%)	5.5 (±13.5)	10.4 (±8.1)^a^	14.3 (±14.6)^a^	<0.001
PPD 4–5 mm (*n*)	–	7.0 (±6.7)	19.2 (±18.8)	<0.001
PPD ≥ 6 mm (*n*)	–	–	1.3 (±2.2)	–

*BOP, bleeding on probing; PPD, probing pocket depth*.

* *Kruskal–Wallis/Mann–Whitney test or Chi-square test*.

a *p < 0.05 in comparison to healthy/gingivitis group*.

b *p < 0.05 in comparison to periodontitis stage I/II group*.

## Discussion

S100A12 is a potent trigger of inflammatory processes and a potential biomarker for chronic inflammatory diseases. However, a detailed characterization of S100A12 kinetics in mononuclear myeloid cells in homeostasis vs. inflammation is lacking. The present study investigated S100A12 dynamics in monocyte subsets, during monocyte maturation stages and polarization, as well as its association with periodontitis. We found that the expression of S100A12 was higher in classical than in non-classical monocytes, decreased during monocyte-to-macrophage differentiation, and was not influenced by macrophage polarization. Peripheral monocytes from periodontitis patients showed altered expression of S100A12. Moreover, in a 3D oral tissue culture inflammatory stimuli modulated S100A12 expression in the presence of monocytes, and the frequency of S100A12^+^ monocyte-derived cells was increased in inflamed gingival tissue affected by periodontitis. Levels of S100A12 in saliva were higher in patients with severe stages of periodontitis. These findings indicate that S100A12 is involved in periodontitis pathogenesis.

We found that classical monocytes (CD14^hi^CD16^−^) exhibited increased S100A12 expression in comparison with non-classical monocytes (CD14^+^CD16^hi^), and the intermediate subset (CD14^hi^CD16^+^) had an expression more similar to the classical subset. This finding is in agreement with previous studies showing higher S100A12 gene expression in the classical subset (3, 6, 30), highlighting its ability to support inflammation (6, 12). Conflicting results have been reported regarding a closer relationship of the intermediate subset to the classical or non-classical subset (5, 6). In our study, S100A12 protein expression was 1.16 times higher in classical monocytes than in intermediates and 1.92 times higher in intermediates than in non-classical monocytes, though no significant difference was seen between the intermediate and the non-classical subsets. Wong et al. (6) have shown that S100A12 gene expression is 13.76 times higher in the classical subset and 2.35 lower in the non-classical subset both in relation to the intermediate subset.

Monocyte-to-macrophage differentiation is a complex process, where major changes occur to the cell global transcriptome (31). Here we show that S100A12 was downregulated during this process under CSF-1 and its expression was not significantly affected when macrophages were later classically or alternatively activated. In agreement with our findings, Sander et al. (32) have shown that monocytes differentiated by CSF-1 had lower expression of S100A12 in comparison with CD14^+^ monocytes. Similarly, Shah et al. (11) have found decreased expression of S100A12 during maturation of monocytes to macrophages along with unaltered expression following polarization. Thus, S100A12 is part of the genes that are rapidly regulated during differentiation, maintained in mature macrophages, and refractory to polarization (31). We have now added that in concordance with gene expression, S100A12 secretion is also reduced during monocyte-to-macrophage differentiation. A decrease in the S100A12 expression during monocyte to macrophage maturation goes in line with lower expression in non-classical monocytes, since both macrophages and circulating non-classical monocytes represent a more advanced stage of differentiation (3, 4).

Interestingly, we found that circulating monocytes from periodontitis patients have an altered expression of S100A12, which was mainly seen in the intermediate and non-classical subsets. The monocytes from periodontitis patients also secreted significantly higher levels of S100A12 after 24 h in monoculture than monocytes from periodontally healthy participants. Similarly, increased expression of S100A12 has been reported in PBMCs from patients with pre-mature coronary artery disease (33). Higher release of prostaglandin E2 and IL-8 has been shown in LPS-stimulated whole blood cell cultures from periodontitis patients in comparison with healthy participants, indicating an intrinsic characteristic or differential priming of the monocytes in periodontitis (34). As S100A12 gene expression is very sensitive to low levels of LPS (12), we speculate that higher exposure to LPS in periodontitis might be at least partially responsible for the enhanced S100A12 expression seen in this study. In fact, transcriptome analysis of circulating monocytes in periodontitis evidenced they might be more functionally active, with the identification of several genes that interact with invading microorganisms or respond to LPS stimulation (35).

Despite being downregulated during monocyte differentiation, a time-dependent increase in S100A12 levels was seen in a 3D oral mucosa culture in the presence of monocytes, which was further increased by LPS and IFN-γ stimulation. Such an increase was not seen in the absence of monocytes, suggesting monocytes are needed for the S100A12 response to inflammatory stimuli in the model. Furthermore, conditioned medium from cultures lacking monocytes did not increase S100A12 secretion by monocytes. It is worth noting that the secreted levels in the cultures were somewhat similar to that of differentiated macrophages in monoculture. Our group previously reported that monocyte-derived cells in the model are CD64^+^ and display increased expression of CD14, CD68, and CD163 during their differentiation, evidencing a macrophage-like phenotype (20). Previous reports have shown that both TLR ligands and IFN-γ induce S100A12 expression in monocytes and/or macrophages (12, 36, 37). S100A12 produced by stimulated monocytes/macrophages might be important for leukocyte recruitment during inflammation (12).

In consonance with the increased expression of S100A12 in peripheral monocytes in periodontitis, we also found higher expression of S100A12 as well as increased frequency of S100A12^+^ monocyte-derived cells in gingival tissue affected by periodontitis. This could be a reflection of increased recruitment of S100A12^+^ monocytes to the inflamed tissue, especially intermediate and non-classical, already altered in peripheral circulation. S100A12 expression is higher in several chronic inflammatory diseases, such as rheumatoid arthritis and inflammatory bowel disease (14–16). S100A12 is a chemoattractant to monocytes (12) and induce a strong inflammatory response in these cells acting as an endogenous TLR4 ligand, which could contribute to the amplification of the inflammatory response (13). Furthermore, S100A12^+^ cells in periodontitis showed a lower expression of CD206 than in controls, and the percentage of S100A12^+^ cells correlated negatively with that of CD206^+^ cells. Viniegra et al. (38) have shown that the expression of CD206, a marker of alternatively activated macrophages, increased during the healing phase of periodontal disease and that the induction of resolving macrophages through a peroxisome proliferator-activated receptor γ (PPAR-γ) agonist reduced the alveolar bone loss and increased CD206 expression in periodontal tissues. Interestingly, a PPAR-γ agonist was shown to inhibit S100A12 expression by macrophages (39), indicating that S100A12 is probably part of the network of inflammatory mediators orchestrating the destruction of periodontal tissues.

Lastly, we found that salivary levels of S100A12 were related to the degree of gingival inflammation and the presence of pathological periodontal pockets. Also, S100A12 levels were significantly higher in patients having periodontitis stages III and IV than in non-periodontitis participants. It is worth mentioning we used radiographic bone loss in the clinical classification instead of clinical attachment loss, which is the primary stage determinant (27). These results are partially in agreement with a previous report from our group, showing that S100A12 alterations were related to gingival bleeding, but not to periodontal pockets, which could be due to the different clinical categorizations used in both cohorts. S100A12 was also related to increased burden of periodontal inflammation (22). According to these reports bleeding seems to be the main determinant of S100A12 levels in saliva and this might be related to the increased density of inflammatory cells in sites with bleeding (40). Taken together, these results highlight S100A12 as a possible biomarker of periodontal inflammation. Whether it has the ability to predict future periodontal destruction deserves further investigation.

In conclusion, S100A12 is predominantly secreted by monocytes rather than by differentiated macrophages. However, S100A12 accumulates in inflamed tissue, potentially as a result of increased production by monocyte-derived cells. This study implicates the involvement of S100A12 in the pathogenesis of periodontitis, and both gingival tissue and circulatory monocytes are altered in periodontitis. S100A12 levels in saliva reflect the severe stages of periodontitis.

## Data Availability Statement

The datasets generated for this study are available on request to the corresponding author.

## Ethics Statement

This study was carried out in accordance with the recommendations of the regional ethics committee in Stockholm, Sweden and of the regional ethics committee at the Lund University, Sweden with written informed consent from all subjects. All subjects gave written informed consent in accordance with the Declaration of Helsinki. The protocol was approved by regional ethics committee in Stockholm, Sweden (Dnr. 2012/1579-32 and 2017/1333-32) and by the regional ethics committee at the Lund University, Sweden (Dnr. 2011/366).

## Author Contributions

RL-J, SH, and EB designed the study, collected and analyzed data, and wrote the manuscript. RC, SZ, and MM collected and analyzed data, and revised the manuscript. GJ, BA, and SÅ collected and interpreted data, and revised the manuscript. MS supervised the experiments. MS and BK contributed to the study design and revised the manuscript. All authors gave final approval of the manuscript.

### Conflict of Interest

The authors declare that the research was conducted in the absence of any commercial or financial relationships that could be construed as a potential conflict of interest.
