# Supplementary figures and images for: S100A12 Expression Is Modulated During Monocyte Differentiation and Reflects Periodontitis Severity
Source: Front Immunol. 2020 Jan 31;11:86. doi: 10.3389/fimmu.2020.00086 (PMC7005221; doi:10.3389/fimmu.2020.00086)

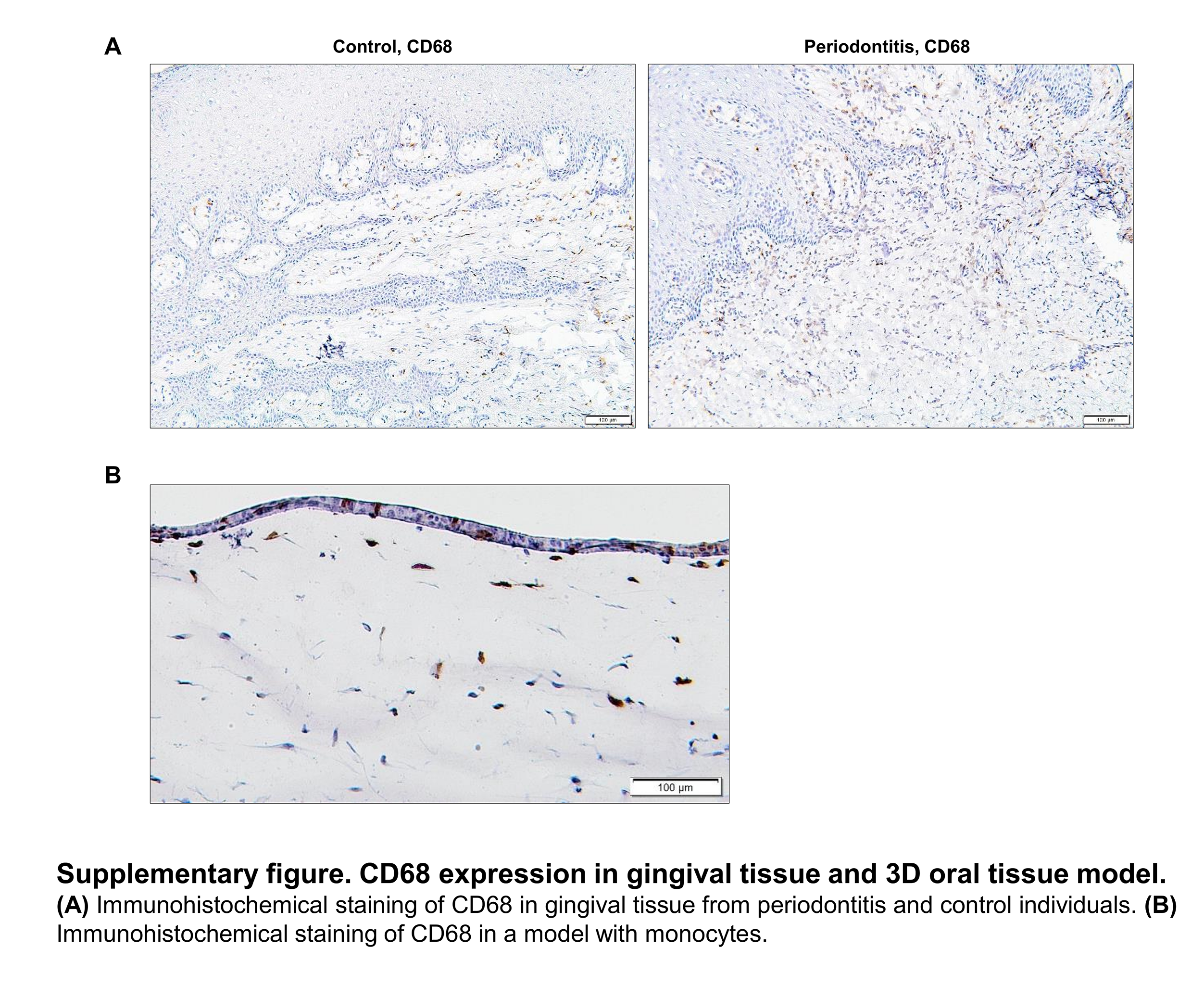

Supplement: Supplementary file 1 [file Image_1.TIF]
